# Molecular targets of vortioxetine mediating glioblastoma suppression revealed by gene and protein network analyses and molecular docking simulations

**DOI:** 10.1093/ijnp/pyaf029

**Published:** 2025-05-02

**Authors:** Chuanjun Zhuo, Chao Li, Qiuyu Zhang, Lei Yang, Ying Zhang, Ximing Chen, Xiaoyan Ma, Ranli Li, Lina Wang, Hongjun Tian

**Affiliations:** Computational Biology and Computational Psychiatry Center (CBCP), Tianjin Anding Hospital, Nankai University Affiliated Tianjin Anding Hospital, Tianjin Medical University Affiliated Tianjin Anding Hospital, Tianjin Mental Health Center of Tianjin Medical University, Tianjin, China; Laboratory of Psychiatric-Neuroimaging-Genetic and Co-morbidity (PGNP_Lab), Tianjin Anding Hospital, Nankai University Affiliated Tianjin Anding Hospital, Tianjin Mental Health Center of Tianjin Medical University, Tianjin, China; Computational Biology and Computational Psychiatry Center (CBCP), Tianjin Anding Hospital, Nankai University Affiliated Tianjin Anding Hospital, Tianjin Medical University Affiliated Tianjin Anding Hospital, Tianjin Mental Health Center of Tianjin Medical University, Tianjin, China; Laboratory of Psychiatric-Neuroimaging-Genetic and Co-morbidity (PGNP_Lab), Tianjin Anding Hospital, Nankai University Affiliated Tianjin Anding Hospital, Tianjin Mental Health Center of Tianjin Medical University, Tianjin, China; Computational Biology and Computational Psychiatry Center (CBCP), Tianjin Anding Hospital, Nankai University Affiliated Tianjin Anding Hospital, Tianjin Medical University Affiliated Tianjin Anding Hospital, Tianjin Mental Health Center of Tianjin Medical University, Tianjin, China; Laboratory of Psychiatric-Neuroimaging-Genetic and Co-morbidity (PGNP_Lab), Tianjin Anding Hospital, Nankai University Affiliated Tianjin Anding Hospital, Tianjin Mental Health Center of Tianjin Medical University, Tianjin, China; Computational Biology and Computational Psychiatry Center (CBCP), Tianjin Anding Hospital, Nankai University Affiliated Tianjin Anding Hospital, Tianjin Medical University Affiliated Tianjin Anding Hospital, Tianjin Mental Health Center of Tianjin Medical University, Tianjin, China; Laboratory of Psychiatric-Neuroimaging-Genetic and Co-morbidity (PGNP_Lab), Tianjin Anding Hospital, Nankai University Affiliated Tianjin Anding Hospital, Tianjin Mental Health Center of Tianjin Medical University, Tianjin, China; Computational Biology and Computational Psychiatry Center (CBCP), Tianjin Anding Hospital, Nankai University Affiliated Tianjin Anding Hospital, Tianjin Medical University Affiliated Tianjin Anding Hospital, Tianjin Mental Health Center of Tianjin Medical University, Tianjin, China; Laboratory of Psychiatric-Neuroimaging-Genetic and Co-morbidity (PGNP_Lab), Tianjin Anding Hospital, Nankai University Affiliated Tianjin Anding Hospital, Tianjin Mental Health Center of Tianjin Medical University, Tianjin, China; Computational Biology and Computational Psychiatry Center (CBCP), Tianjin Anding Hospital, Nankai University Affiliated Tianjin Anding Hospital, Tianjin Medical University Affiliated Tianjin Anding Hospital, Tianjin Mental Health Center of Tianjin Medical University, Tianjin, China; Laboratory of Psychiatric-Neuroimaging-Genetic and Co-morbidity (PGNP_Lab), Tianjin Anding Hospital, Nankai University Affiliated Tianjin Anding Hospital, Tianjin Mental Health Center of Tianjin Medical University, Tianjin, China; Computational Biology and Computational Psychiatry Center (CBCP), Tianjin Anding Hospital, Nankai University Affiliated Tianjin Anding Hospital, Tianjin Medical University Affiliated Tianjin Anding Hospital, Tianjin Mental Health Center of Tianjin Medical University, Tianjin, China; Laboratory of Psychiatric-Neuroimaging-Genetic and Co-morbidity (PGNP_Lab), Tianjin Anding Hospital, Nankai University Affiliated Tianjin Anding Hospital, Tianjin Mental Health Center of Tianjin Medical University, Tianjin, China; Computational Biology and Computational Psychiatry Center (CBCP), Tianjin Anding Hospital, Nankai University Affiliated Tianjin Anding Hospital, Tianjin Medical University Affiliated Tianjin Anding Hospital, Tianjin Mental Health Center of Tianjin Medical University, Tianjin, China; Laboratory of Psychiatric-Neuroimaging-Genetic and Co-morbidity (PGNP_Lab), Tianjin Anding Hospital, Nankai University Affiliated Tianjin Anding Hospital, Tianjin Mental Health Center of Tianjin Medical University, Tianjin, China; Computational Biology and Computational Psychiatry Center (CBCP), Tianjin Anding Hospital, Nankai University Affiliated Tianjin Anding Hospital, Tianjin Medical University Affiliated Tianjin Anding Hospital, Tianjin Mental Health Center of Tianjin Medical University, Tianjin, China; Laboratory of Psychiatric-Neuroimaging-Genetic and Co-morbidity (PGNP_Lab), Tianjin Anding Hospital, Nankai University Affiliated Tianjin Anding Hospital, Tianjin Mental Health Center of Tianjin Medical University, Tianjin, China; Computational Biology and Computational Psychiatry Center (CBCP), Tianjin Anding Hospital, Nankai University Affiliated Tianjin Anding Hospital, Tianjin Medical University Affiliated Tianjin Anding Hospital, Tianjin Mental Health Center of Tianjin Medical University, Tianjin, China; Laboratory of Psychiatric-Neuroimaging-Genetic and Co-morbidity (PGNP_Lab), Tianjin Anding Hospital, Nankai University Affiliated Tianjin Anding Hospital, Tianjin Mental Health Center of Tianjin Medical University, Tianjin, China

**Keywords:** vortioxetine, glioblastoma, network pharmacology, molecular docking

## Abstract

**Background:**

Vortioxetine is a serotonin reuptake inhibitor and serotonin receptor modulator used for the treatment of major depressive disorder, but recent studies have also reported anticancer effects in models of glioblastoma. Given the well-established benefits of drug repositioning, we examined the pharmacological mechanism for these anticancer actions using bioinformatics and molecular docking.

**Methods:**

Putative molecular targets for vortioxetine were identified by searching DrugBank, GeneCards, SwissTargetPrediction, Comparative Toxicogenomics Database, and SuperPred databases, while glioblastoma-related proteins were identified using GeneCards, Online Mendelian Inheritance in Man; , and Therapeutic Target Database . A protein–protein interaction (PPI) network was constructed from vortioxetine targets also involved in glioblastoma to identify core (hub) targets, which were then characterized by Gene Ontology (GO) and Kyoto Encyclopedia of Genes and Genomes (KEGG) pathway enrichment analyses using database for annotation, visualization, and integrated discovery. Cytoscape was utilized to generate a drug-pathway-target-disease network, and molecular docking simulations were performed to evaluate direct interactions between vortioxetine and core target proteins.

**Results:**

A total of 234 unique vortioxetine protein targets were identified. Among 234 vortioxetine targets identified, 48 were also related to glioblastoma. Topological analysis of the PPI network revealed 5 core targets: the serine/threonine kinase AKT1, transcription factor hypoxia-inducible factor (HIF)-1, cell adhesion molecule cadherin-E, NF-κB subunit p105, and prostaglandin-endoperoxide synthase 2. According to GO and KEGG pathway analyses, the anticancer efficacy of vortioxetine may be mediated by effects on glucose metabolism, cell migration, phosphorylation, inflammatory responses, apoptosis, and signaling via Rap1, chemical carcinogenesis-reactive oxygen species, and HIF-1. Molecular docking revealed moderately strong affinities between vortioxetine and 4 core targets.

**Conclusions:**

This study suggests that vortioxetine may inhibit glioblastoma development through direct effects on multiple targets and further emphasizes the value of bioinformatics analyses for drug repositioning.

**Significance Statement**

Glioblastoma remains one of the most aggressive and treatment-resistant brain tumors, with limited success from conventional therapies. Given the challenges in developing novel treatments, drug repurposing has emerged as a promising alternative. This study identifies AKT1, HIF1A, CDH1, and NFKB1 as potential targets of vortioxetine, a clinically approved antidepressant, in suppressing glioblastoma. The findings suggest that vortioxetine may exert anticancer effects by regulating glucose metabolism, cell migration, phosphorylation, inflammatory responses, apoptosis, and key signaling pathways such as Rap1, chemical carcinogenesis-reactive oxygen species, and HIF-1. These results not only enhance our understanding of vortioxetine’s anticancer potential but also highlight the broader significance of repurposing BBB-permeable neuroactive drugs for intracranial tumors. By integrating bioinformatics and molecular docking, this study provides a theoretical foundation for further experimental validation and clinical translation, contributing to the development of more effective glioblastoma treatments.

## INTRODUCTION

Glioblastoma accounts for almost half of all primary brain tumors in adults.^1,2^ It is also classified as the most aggressive grade IV tumor type by the World Health Organization according to histopathologic features such as necrosis and endothelial proliferation.^3^ Glioblastoma is almost invariably lethal, with a median survival of only about 15 months following surgery and adjuvant chemoradiotherapy^4^ and a 5-year survival rate of only 5% to 10%^5-7^ despite being the most well-funded disease for research among intracranial malignancies by the United States National Institutes of Health over the past 50 years.^8^ Complete surgical resection is limited by the highly invasive nature of glioblastoma cells, while multimodal non-surgical therapies such as temozolomide and radiation frequently fail due to the development of resistance.^9^ Moreover, targeted therapies have shown limited success in clinical trials, partly due to the restricted tumor accessibility imposed by the blood-brain barrier (BBB). Addressing these therapeutic roadblocks remains an urgent clinical need.

The development of novel small molecules for human cancer treatment is a lengthy and costly process involving multiple stages of preclinical investigations and clinical trials. In contrast, repurposing or repositioning available drugs for new therapeutic indications is generally more cost-effective and time-efficient as side effects and toxicity profiles are already established.^9^ Two recent studies reported that the antidepressant vortioxetine can suppress glioblastoma cell proliferation.^10,11^ Vortioxetine is a multimodal antidepressant designed to inhibit serotonin reuptake and modulate multiple neurotransmitter systems through interactions with various serotonin receptors (including 5-HT_1A_, 5-HT_1B_, 5-HT_1D_, 5-HT_3_, and 5-HT_7_).^12^ A meta-analysis concluded that vortioxetine is as effective as specific serotonin reuptake inhibitors (SSRIs) and serotonin–norepinephrine reuptake inhibitors (SNRIs) for treating major depressive disorder but with a safety profile superior to SNRIs and comparable to SSRIs.^13^ Vortioxetine has also demonstrated efficacy for treating depression-associated symptoms such as anhedonia, emotional blunting, anxiety, and cognitive dysfunction.^14^ However, the molecular mechanisms underlying these effects, including those against glioblastoma, remain unclear.

Network pharmacology, a concept first introduced by Hopkins,^15^ has emerged as a promising approach for elucidating the action mechanisms of existing drugs and for discovering new therapeutic agents. With advances in bioinformatics, network pharmacology has become a powerful tool for computationally predicting and systematically validating the molecular mechanisms underlying complex diseases and drug responses. These findings can be further supported by molecular docking, a virtual screening technique for analyzing the interactions between protein receptors and small molecules.

**Figure 1.**
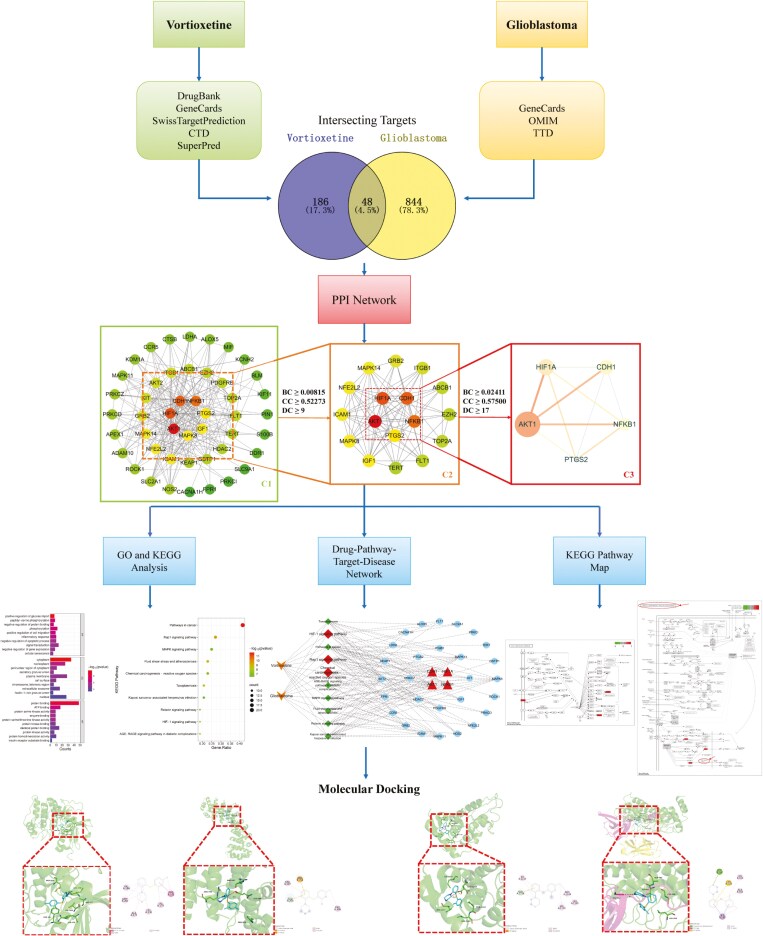
Workflow of the study.

Our recent studies have applied network pharmacology and molecular docking techniques to explore the potential mechanisms behind the therapeutic effects of quetiapine on bipolar disorder and fingolimod on cognitive impairment associated with schizophrenia.^16,17^ In the current study, we utilized network pharmacology and molecular docking to explore the potential molecular mechanisms underlying the suppressive effects of vortioxetine on glioblastoma. These findings are expected to provide new perspectives on the repurposing of BBB-permeable neuroactive drugs for intracranial tumors and offer theoretical guidance for further clinical trials and the design of new drugs. The workflow is shown in Figure 1.

## METHODS

### Identification of Vortioxetine Protein Targets and Glioblastoma-Associated Proteins

The chemical structure of vortioxetine was obtained from the DrugBank database (https://go.drugbank.com),^18^ and target proteins were predicted by searching DrugBank, GeneCards (https://www.genecards.org/),^19^ SwissTargetPrediction (http://www.swisstargetprediction.ch/),^20^ Comparative Toxicogenomics Database (CTD) (https://ctdbase.org/),^21^ and SuperPred (https://prediction.charite.de/)^22^ databases using “vortioxetine” as the keyword. Duplicates were removed by manually searching the lists retrieved. Proteins associated with glioblastoma were identified by searching GeneCards, Online Mendelian Inheritance in Man (OMIM) (https://www.omim.org/)^,23^ and Therapeutic Target Database (TTD) (https://db.idrblab.net/ttd/)^24^ using “glioblastoma” as the keyword. Again, duplicates were removed by manually searching the lists retrieved. The Venn diagram program Venny 2.1.0 (https://bioinfogp.cnb.csic.es/tools/venny) was then used to identify molecules common to both (duplicate-free) vortioxetine target and glioblastoma-related protein lists.

### Protein–Protein Interaction Network Construction and Core Target Screening

The common targets were uploaded to the STRING database (https://string-db.org) for the construction of a protein–protein interaction (PPI) network.^25^ The analysis was restricted to *Homo sapiens* and associations with a confidence score ≥ 0.4. Disconnected nodes were excluded. The topological properties of the PPI network were then analyzed using Cytoscape 3.10.0 (https://www.cytoscape.org/).^26^ Three key topological parameters, degree centrality (DC), betweenness centrality (BC), and closeness centrality (CC), were calculated to identify core targets, with median DC, BC, and CC values set as the cutoffs for screening. The MCODE plug-in of Cytoscape was employed to identify clusters in the PPI network according to the following parameters: degree cutoff = 2, node score cutoff = 0.2, K-core = 2, and maximum depth = 100.

### Gene Ontology and Kyoto Encyclopedia of Genes and Genomes Enrichment Analyses

The cellular and signaling functions of common targets were then investigated by Gene Ontology (GO) and Kyoto Encyclopedia of Genes and Genomes (KEGG) enrichment analyses using the Database for Annotation, Visualization, and Integrated Discovery (https://david.ncifcrf.gov/).^27^ The top 10 significantly enriched GO terms within the biological process (BP), cellular component (CC), and molecular function (MF) categories (all *P* < .05), and the top 10 KEGG pathways (*P* < .05) were selected for further analyses. The results were visualized using the Bioinformatics platform (http://www.bioinformatics.com.cn).

### Drug-Pathway-Target-Disease Network Construction

A drug-target-pathway-disease network was constructed and analyzed using Cytoscape 3.10.0 to identify and visualize the core targets and pathways mediating the effects of vortioxetine on glioblastoma.

### Molecular Docking Simulations

Molecular docking simulations were performed using AutoDockTools 1.5.6 and AutoDock Vina^28^ to predict the binding affinities of vortioxetine with core targets. The workflow included 4 steps.

### The First Step is Ligand Preparation

First, the sdf structure file of vortioxetine was downloaded from the PubChem database (https://pubchem.ncbi.nlm.nih.gov/)^29^ and converted into a 3D structure based on energy minimization using ChemBio3D Ultra 14.0. This 3D structure was then converted to pdbqt file format using AutoDockTools 1.5.6.

### The Second Step is Protein Preparation

In the second step, the crystal structures of core target proteins were obtained from the Protein Data Bank (http://www.rcsb.org) using gene symbols.^30^ Water molecules and initial ligands were removed using PyMOL 2.4.1, and then hydrogenation, charge calculations, and atom type assignments were conducted using AutoDockTools 1.5.6. Files were again saved in pdbqt format.

### The Third Step is Grid Preparation

The third step involved grid preparation, where the docking box size and coordinates were adjusted based on potential binding sites identified using DoGSiteScorer (https://proteins.plus/).

### The Fourth Step is Molecular Docking

Molecular docking and binding energy evaluation were performed using AutoDock Vina. Finally, PyMOL 2.4.1 and Discovery Studio 2021 were used to visualize and analyze the interactions between vortioxetine and the core target proteins.

Table 1 presents an overview of the tools and resources employed.

**Table 1.** Project overview and resources.

Project	Databases and tools	Source
Target collection	DrugBank	https://go.drugbank.com
	GeneCards	https://www.genecards.org/
	CTD	https://ctdbase.org/
	SuperPred	https://prediction.charite.de/
	OMIM	https://www.omim.org/
	TTD	https://db.idrblab.net/ttd/
Drug–disease target intersection	STRING	https://string-db.org
	Venny 2.1.0	https://bioinfogp.cnb.csic.es/tools/venny
	Cytoscape 3.10.0	https://www.cytoscape.org/
	Cytoscape MCODE plug-in	https://www.cytoscape.org/
GO and KEGG enrichment analysis	DAVID	https://david.ncifcrf.gov/
	Bioinformatics platform	http://www.bioinformatics.com.cn/
Drug-pathway-target-disease network	Cytoscape 3.10.0	https://www.cytoscape.org/
Molecular docking	PubChem	https://pubchem.ncbi.nlm.nih.gov/
	AutoDockTools 1.5.6	https://autodock.scripps.edu/
	AutoDock Vina	https://vina.scripps.edu/
	PDB	http://www.rcsb.org
	DoGSiteScorer	https://proteins.plus/
	PyMOL 2.4.1	https://pymol.org/
	Discovery Studio 2021	https://www.3ds.com/products/biovia/discovery-studio

Abbreviations: CTD, Comparative Toxicogenomics Database; DAVID, Database for Annotation, Visualization, and Integrated Discovery; OMIM, Online Mendelian Inheritance in Man; PDB, Protein Data Bank; TTD, Therapeutic Target Database.

## RESULTS

### Identification of Vortioxetine Protein Targets Associated with Glioblastoma

**Figure 2.**
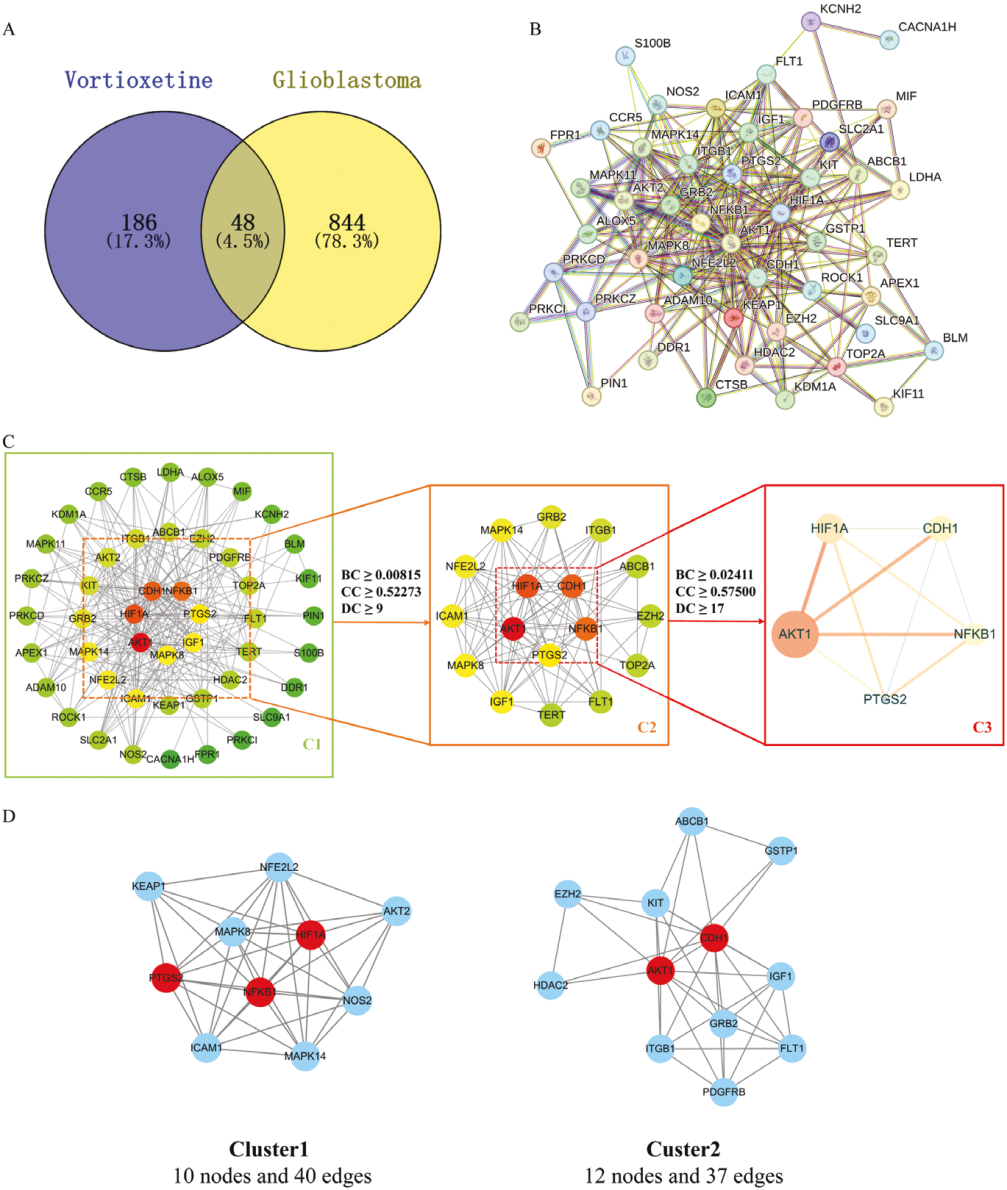
**Identification of core vortioxetine targets also involved in glioblastoma via protein–protein interaction network analysis. A**: Venn diagram showing vortioxetine target proteins also implicated in glioblastoma. **B**: PPI network diagram of common targets obtained from the STRING database. **C**: Core targets identified among these common targets by applying critical topological characteristic thresholds (DC, BC, and CC). **D**: Cluster analysis of the PPI network using the MCODE plug-in. **Abbreviations:** BC, betweenness centrality; CC, closeness centrality; DC, degree centrality.

Screening of the DrugBank, GeneCards, SwissTargetPrediction, CTD, and SuperPred databases identified 7, 31, 108, 8, and 121 vortioxetine targets, respectively. After eliminating duplicates, a total of 234 unique vortioxetine protein targets were identified. Similarly, screening the GeneCards, OMIM, and TTD databases yielded 859, 13, and 59 proteins associated with glioblastoma, respectively, for a total of 892 non-redundant proteins. A Venn diagram revealed 48 vortioxetine targets also associated with glioblastoma (Figure 2A).

### PPI Network Construction and Core Target Identification

A PPI network was then constructed from these 48 proteins using the STRING database (Figure 2B). The initial PPI network consisted of 47 nodes and 255 edges (Figure 2C1). Subsequently, a refined network comprising 17 target nodes and 85 edges was obtained based on the criteria of DC, BC, and CC ≥ median values from Figure 2C1 (Figure 2C2). Finally, applying the same criteria to Figure 2C2, 5 core targets and 10 edges were identified (Figure 2C3). Additionally, clustering analysis of the PPI network using the MCODE plug-in of Cytoscape revealed 2 subnetworks (Figure 2D). The 5 core targets were the serine/threonine kinase AKT1, the transcription factor hypoxia-inducible factor (HIF)-1, the cell adhesion molecule cadherin-1, the stress-activated transcription factor NF-κB p105 subunit, and prostaglandin-endoperoxide synthase 2 (also known as cyclo-oxygenase 2 or COX-2).

### GO Enrichment Analysis

**Figure 3.**
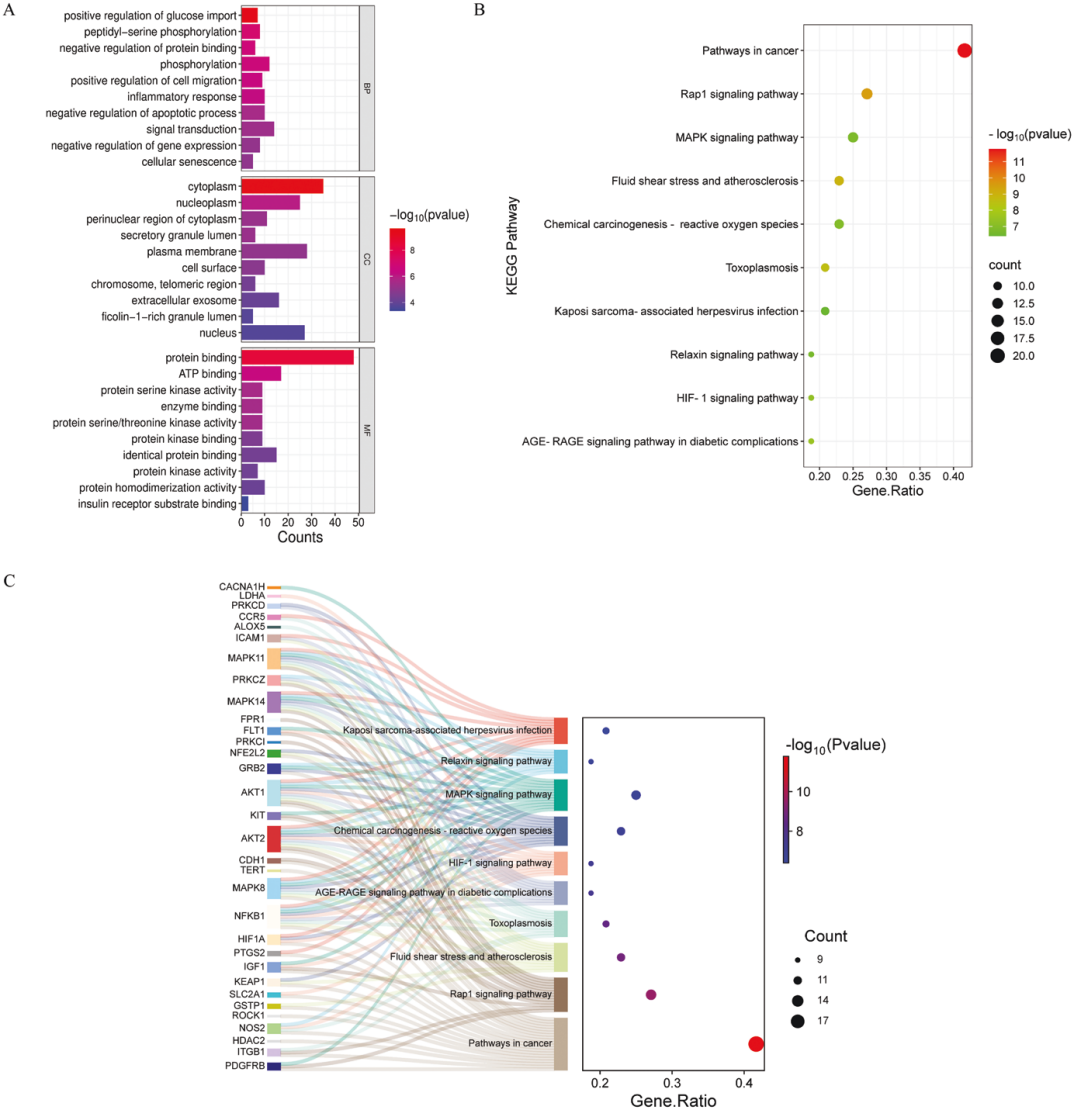
**GO and KEGG pathway enrichment analyses. A**: Bar charts showing the top 10 enriched BPs, CCs, and MFs from GO enrichment analysis. **B**: Bubble charts of KEGG pathway enrichment analyses showing the top 10 enriched pathways for core genes. **C**: Sankey diagram based on KEGG enrichment analysis. The left rectangular nodes of the Sankey diagram represent the therapeutic targets, the right nodes represent the KEGG pathways, and the lines represent the associations between the targets and pathways. In (B) and (C), counts are indicated by symbol size and *P*-values by color. **Abbreviates:** BPs, biological processes; CCs, cellular components; GO, Gene Ontology, KEGG, Kyoto Encyclopedia of Genes and Genomes; MFs, molecular functions.

Gene Ontology enrichment analysis was then performed to investigate the molecular mechanisms mediating the effects of vortioxetine on glioblastoma. A total of 155 BPs, 34 CCs, and 31 MFs were identified. The top 10 terms for each GO category are presented in a column chart (Figure 3A). Highly enriched BP annotations included “positive regulation of glucose import,” “cell migration,” “phosphorylation,” “inflammatory response,” and “apoptotic process,” enriched CCs included “cytoplasm,” “nucleoplasm,” “secretory granule lumen,” and “chromosome telomeric region,” and enriched MFs included “protein binding,” “ATP binding,” and “protein serine/threonine kinase activity.”

### KEGG Pathway Analysis and Drug-Pathway-Target-Disease Network Construction

**Figure 4.**
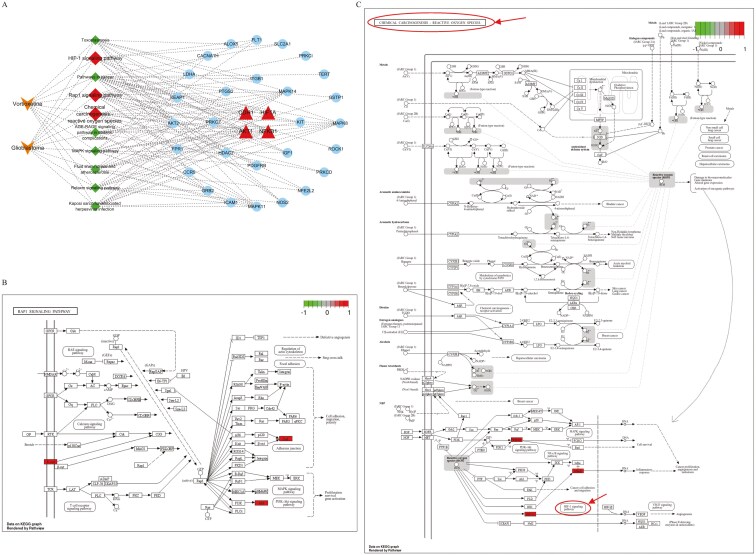
**Drug-pathway-target-disease network diagrams and distribution of core targets in the main pathways. A**: Drug-pathway-target-disease network diagram for the effects of vortioxetine on glioblastoma. **B**: Distribution of the vortioxetine and glioblastoma overlapping hub genes in the Rap1 signaling pathway. **C**: Distributions of the vortioxetine and glioblastoma overlapping hub genes in the chemical carcinogenesis-reactive oxygen species and HIF-1 signaling pathways.

The 48 common targets were significantly enriched in 109 pathways, of which the top 10 are presented in Table 2 and Figure 3B, while connections between target genes and the top 10 enriched pathways are presented as a Sankey diagram in Figure 3C. To show the positions of core targets in key signaling pathways, we also present diagrams of the Rap1, chemical carcinogenesis-reactive oxygen species (ROS), and HIF-1 signaling pathways (Figure 4B and C). The established drug-pathway-target-disease network diagram depicting the effects of vortioxetine on glioblastoma included 44 nodes (1 drug, 10 pathway, 32 target, and 1 disease node) and 91 edges (Figure 4A).

**Table 2.** KEGG enrichment analysis of intersecting targets.

ID	Term	Genes	Count	*P*-value
hsa05200	Pathways in cancer	PDGFRB, ITGB1, HDAC2, NOS2, ROCK1, GSTP1, SLC2A1, KEAP1, IGF1, PTGS2, HIF1A, NFKB1, MAPK8, TERT, CDH1, AKT2, KIT, AKT1, GRB2, NFE2L2	20	1.67E-12
hsa04015	Rap1 signaling pathway	PDGFRB, ITGB1, PRKCI, FLT1, FPR1, IGF1, MAPK14, PRKCZ, MAPK11, CDH1, AKT2, KIT, AKT1	13	2.85E-10
hsa05418	Fluid shear stress and atherosclerosis	MAPK11, MAPK8, AKT2, GSTP1, KEAP1, AKT1, MAPK14, PRKCZ, NFKB1, ICAM1, NFE2L2	11	1.21E-09
hsa05145	Toxoplasmosis	ITGB1, MAPK11, MAPK8, NOS2, AKT2, ALOX5, AKT1, CCR5, MAPK14, NFKB1	10	2.75E-09
hsa04933	AGE-RAGE signaling pathway in diabetic complications	MAPK11, MAPK8, AKT2, PRKCD, AKT1, MAPK14, PRKCZ, NFKB1, ICAM1	9	2.77E-08
hsa04066	HIF-1 signaling pathway	LDHA, FLT1, NOS2, AKT2, SLC2A1, AKT1, IGF1, HIF1A, NFKB1	9	5.06E-08
hsa05208	Chemical carcinogenesis-reactive oxygen species	MAPK11, MAPK8, AKT2, PRKCD, KEAP1, AKT1, GRB2, MAPK14, HIF1A, NFKB1, NFE2L2	11	1.13E-07
hsa04010	MAPK signaling pathway	PDGFRB, MAPK11, MAPK8, FLT1, AKT2, KIT, AKT1, GRB2, IGF1, MAPK14, CACNA1H, NFKB1	12	1.62E-07
hsa04926	Relaxin signaling pathway	MAPK11, MAPK8, NOS2, AKT2, AKT1, GRB2, MAPK14, PRKCZ, NFKB1	9	2.00E-07
hsa05167	Kaposi sarcoma-associated herpesvirus infection	MAPK11, MAPK8, AKT2, AKT1, CCR5, MAPK14, PTGS2, HIF1A, NFKB1, ICAM1	10	3.91E-07

Abbreviation: KEGG, Kyoto Encyclopedia of Genes and Genomes.

### Molecular Docking

**Figure 5.**
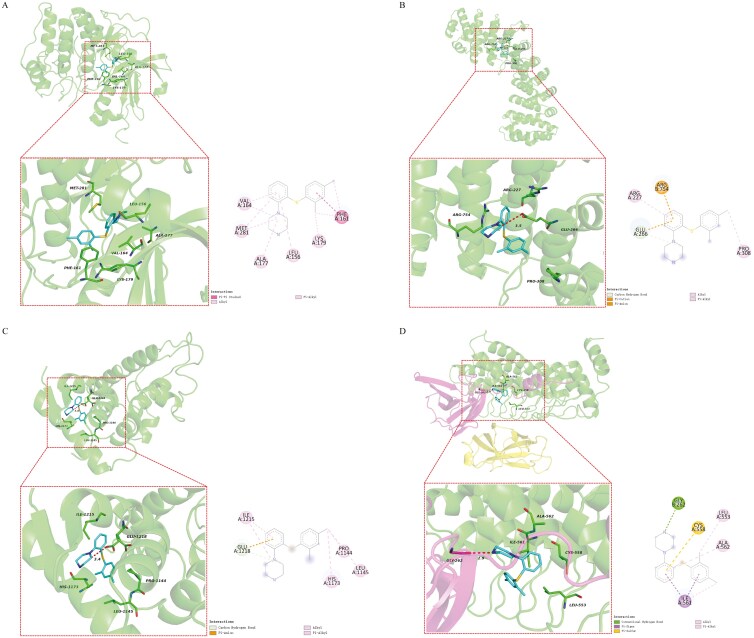
**Molecular docking results for vortioxetine and core targets. A**: Vortioxetine and AKT1. **B**: Vortioxetine and HIF-1. **C**: Vortioxetine and CDH1. **D**: Vortioxetine and NFKB1.

To validate the results of network pharmacology analysis, molecular docking simulations were conducted between vortioxetine and core targets (Figure 5). Based on the results of the PPI network and KEGG enrichment analysis, AKT1, HIF1A, CDH1, and NFKB1 were identified as vortioxetine targets that also regulate glioblastoma. The docking scores for vortioxetine binding to the core targets ranged from − 5.6 to − 10.1 kcal/mol (Table 3), indicating stable binding. The strongest binding affinity was between vortioxetine and AKT1 (− 10.1 kcal/mol) due to hydrophobic interactions at ALA-177, LEU-156, LYS-179, MET-281, PHE-161, and VAL-164. Vortioxetine also exhibited good binding affinity for HIF1A (− 5.6 kcal/mol) through hydrogen bonding at GLU-266, electrostatic interactions with ARG754 and GLU-266, and hydrophobic interactions with ARG-227 and PRO-308. The stronger binding with CDH1 (− 6.8 kcal/mol) was mediated by a hydrogen bond with GLU-1218, electrostatic interactions with GLU-1218, and hydrophobic interactions with HIS-1173, ILE-1215, LEU-1145, and PRO-1144, while binding with NFKB1 (− 6.6 kcal/mol) was mediated by hydrogen bonding at GLY-262 and hydrophobic interactions at ILE-561, CYS-558, LEU-553, and ALA-562, along with additional interactions at CYS-558.

**Table 3.** Molecular docking scores (kcal/mol).

Ligand	Receptor	PDB ID	Docking scores
Vortioxetine	AKT1	3CQU	−10.1
	HIF1A	8HE3	−5.6
	CDH1	4B4C	−6.8
	NFKB1	9BOR	−6.6

Abbreviation: PDB, Protein Data Bank.

## DISCUSSION

Glioblastoma is a devastating disease with a poor prognosis, and current systemic treatments are limited to DNA-alkylating chemotherapies with severe side-effects profiles and high rates of failure. A recent review asserted that antidepressants may have anticarcinogenic effects or act synergistically with conventional anticancer drugs,^31^ suggesting that these and other BBB-permeable neuroactive agents are a potential source of novel cancer treatments. Indeed, recent studies using this drug repurposing strategy have verified the suppressive effects of vortioxetine on glioblastoma cell growth,^10,11^ although the underlying mechanisms of action remain unclear. Therefore, we used network pharmacology and molecular docking approaches to explore the potential mechanisms for glioblastoma suppression by vortioxetine. A multi-level drug-pathway-target-disease network integrating structural and functional information on known vortioxetine-binding targets and proteins associated with glioblastoma identified several promising potential anticarcinogenic targets of vortioxetine.

Screening for vortioxetine-binding proteins and glioblastoma-associated proteins revealed 48 common proteins, and bioinformatics analysis of the PPI network constructed from these proteins identified 5 core targets with potentially broad effects on glioblastoma-associated pathways. Of these, AKT1, HIF1A, CDH1, and NFKB1 were chosen for detailed bioinformatics analysis and vortioxetine-binding simulations.

The AKTs are a family of 3 serine/threonine kinases (AKT1, AKT2, and AKT3) with numerous protein targets and diverse functions in cell proliferation, survival, migration, and metabolism.^32,33^ Overexpression of AKT1 is frequently observed in breast cancer, liver cancer, and glioblastoma,^34-36^ and higher overexpression has been reported in glioblastoma patients with poorest prognosis.^35,37^ Although glioblastoma is one of the most vascularized tumors in humans, the microcirculation is highly inefficient, leading to areas of severe hypoxia and necrosis. The transcription factor HIF-1 is the primary mediator of the protective response to hypoxia and thus essential for tumor survival and progression^.38^ Once HIF-1 enters the nucleus, it activates the transcription of over a hundred target genes, including genes involved in angiogenesis, invasion, glucose metabolism, and lipid metabolism.^39,40^ Thus, suppression of HIF-1 transactivation activity by vortioxetine may enhance hypoxic death of glioblastoma cells and thereby limit disease progression.

The CDH1 gene encodes cadherin-E, a cell adhesion protein also implicated in tumor suppression. Methylation of *CDH1* and concomitantly reduced cadherin-E expression has been reported to increase the recurrence rates and invasive potentials of breast cancer and glioblastoma, leading to poorer prognosis.^41-43^ The NFKB1 gene encodes a member of the nuclear factor-kappa B (NF-κB) family of stress-responsive transcription factors. Mice lacking NFKB1 exhibited inflammation, heightened susceptibility to DNA damage, and accelerated aging,^44^ changes known to promote cancer development. The long non-coding RNA LBX2-AS1 activates the proinflammatory interleukin-4 receptor by binding to NFKB1, which in turn enhances glioblastoma metastasis and angiogenesis.^45^ Thus, these core targets can all modulate the risk of glioblastoma through distinct mechanisms. As all bind vortioxetine, this antidepressant holds great potential as a novel multimodal glioblastoma treatment with established safety.

Gene ontology enrichment analyses revealed several BPs that likely are directly associated with the effects of vortioxetine on glioblastoma, including positive regulation of glucose import, cell migration, phosphorylation, inflammatory responses, and apoptosis. Glucose metabolism is essential for cell migration and thus for glioblastoma invasiveness.^46^ Furthermore, programmed cell death alters the inflammatory tumor microenvironment and facilitates glioblastoma progression.^47^ Kyoto Encyclopedia of Genes and Genomes pathway analyses identified Rap1, chemical carcinogenesis-ROS, and HIF-1 signaling as major pathways linking vortioxetine binding to glioblastoma. Results of a cell model study suggested that the protein kinase A (PKA) and Epac1/Rap1 pathways may collaborate to promote rolipram-induced death of A172 human glioblastoma cells.^48^ Alternatively, downstream of kinase 1 was reported to regulate platelet-derived growth factor BB-mediated glioma cell invasion through a p130Cas–Rap1 signaling pathway.^49^ Furthermore, nitric oxide/ROS-scavenging nanoparticles enhanced glioblastoma immunotherapy by reducing T-cell suppression.^50^ The ROS–AMPK–HIF-1α signaling pathway activated by serine and glycine deprivation was found to induce genes involved in glucose uptake, glycolysis, and serine synthesis, and to promote glucose-derived de novo serine and glycine biosynthesis, activities essential for glioblastoma cell proliferation, survival, and growth.^51^ Additionally, cycling hypoxia-induced Bcl-xL expression mediated by ROS-induced HIF-1α and NF-κB activation suppressed apoptosis and promoted glioblastoma chemoresistance.^52^ Of the 5 core targets, 3 (AKT1, HIF1A, and NFKB1) are common to both the chemical carcinogenesis-ROS and HIF-1 signaling pathways, while 2 (AKT1 and CDH1) are involved in the Rap1 signaling pathway according to database searches. Based on these findings, we propose a computational biology-based molecular mechanism for vortioxetine-induced suppression of glioblastoma involving the Rap1, chemical carcinogenesis-ROS, and HIF-1 signaling pathways, and linked to modulation of glucose metabolism, ROS generation, cell migration, inflammation, and apoptosis. Further experimental studies are needed to determine the exact mechanisms.

Finally, molecular docking simulations validated the binding of vortioxetine to these 4 core target proteins (AKT1, HIF1A, CDH1, and NFKB1) and estimated the individual affinities. The docking scores between ligands and proteins ranged from − 5.6 to − 10.1 kcal/mol, indicating that vortioxetine binds with moderately strong affinity to these core target proteins. These results suggest that vortioxetine may target these core proteins to modulate glioblastoma and that antidepressants with similar structural moieties may also possess anticarcinogenic effects.

This study has several limitations. First, the targets identified are only those submitted to online databases, and there may be many as yet undiscovered vortioxetine-binding targets associated with glioblastoma or other cancers. High-throughput screening of other neuroactive agents for effects against glioblastoma cell proliferation, migration, and invasion in vitro is warranted to identify other promising candidates. Second, although the safety of vortioxetine has been established in patients with major depressive disorder, safety at doses effective for glioblastoma requires validation. Finally, this study relied on a computational approach, so further in vivo and in vitro experiments as well as clinical trials are necessary to confirm the molecular mechanisms responsible for the anticarcinogenic effects of vortioxetine.

In conclusion, this study employed a comprehensive bioinformatics approach, including drug–disease target intersection, biological function annotations, and signaling pathway analysis to explore the mechanisms underlying the effects of vortioxetine on glioblastoma. These bioinformatics and computational analyses suggest that vortioxetine suppresses glioblastoma development by modulating the functions and activities of AKT1, HIF1A, CDH1, NFKB1, and associated signaling pathways, such as the Rap1, chemical carcinogenesis-ROS, and HIF-1 signaling pathways. This study also highlights the potential of repurposing BBB-permeable neuroactive drugs for intracranial tumors.

## Data Availability

Datasets generated during the experiments are available from the corresponding author upon reasonable request.
